# The Church and the Marriage of Syphilitics

**Published:** 1919-06-28

**Authors:** E. Ashton Stephens

**Affiliations:** Swansea.


### THE EDITOR'S LETTER-BOX.

**[Correspondence on all subjects is invited, but we cannot in any way be responsible for the opinions expressed by our correspondents, who must give their name and address as a guarantee of good faith, but not necessarily for publication. Correspondents are reminded that brevity of style and conciseness of statement greatly facilitate early insertion.]**

### The Church and the Marriage of Syphilitics.

*To the Editor of* The Hospital.

Sir,—In your very interesting and important article, "Professional Secrecy," you state that any syphilitic person who knowingly marries commits an infamous act.

What of the priest who performs the rite, knowing that, say, the man is infected with syphilis?

I know a priest who did this although I warned him.

Yours,

Swansea. E. Ashton Stephens.

